# Optical coherence tomography angiography in Purtscher-like retinopathy associated with dermatomyositis: a case report

**DOI:** 10.1186/s13256-019-2152-2

**Published:** 2019-07-06

**Authors:** D. Vezzola, D. Allegrini, M. R. Romano, L. Pagano, A. Montericcio, P. Fogagnolo, L. M. Rossetti, S. De Cillà

**Affiliations:** 1 Eye Unit, University Hospital Maggiore della Carita, Novara, Italy; 2 Eye Clinic, Humanitas Castelli, Humanitas Gavazzeni Hospital, Humanitas University, Bergamo, Italy; 3 Eye Clinic, San Paolo Hospital, University of Milan, Milan, Italy

**Keywords:** OCTA, Dermatomyositis, Retinopathy

## Abstract

**Purpose:**

To describe a multimodal imaging diagnosis of retinopathy in dermatomyositis.

**Case presentation:**

A 21-year-old white woman with a history of fatigue and a cutaneous rash complained of visual impairment in her left eye. A funduscopic examination showed multiple confluent cotton-wool spots in both eyes. Swept source-optical coherence tomography presented macular edema in both eyes; optical coherence tomography angiography revealed superficial and deep capillary occlusion in all areas affected by cotton-wool spots; and fluorescein angiography showed vascular walls enhancement, veins dilatation, and capillary leakage. After large doses of intravenously administered glucocorticoid therapy, followed by a cyclophosphamide regimen, best corrected visual acuity returned to 20/20 in both eyes.

**Conclusions:**

This case report presents optical coherence tomography angiography clinical findings in a rare case of dermatomyositis-associated retinopathy, remarking the importance of a multi-imaging approach for a correct diagnosis and treatment of eye injuries, in order to avoid serious complications and permanent sequelae.

## Introduction

Retinopathy associated with dermatomyositis (DM) is rare and was first described by Bruce in 1938 [1], who presented two patients with ill-defined areas of grayish-yellow exudate, roughly circular in shape and varying in size, distended veins, and occasional deep or superficial hemorrhage.

Since then, a few case reports have reported it in adults and children with the same fundus appearance [2–7].

Further studies classified this clinical appearance under the name of Purtscher-like retinopathy associated with DM [8].

Purtscher retinopathy refers to a chorioretinopathy associated with indirect trauma characterized by funduscopic appearance with cotton-wool spots (CWS), retinal hemorrhages, optic disc edema, and Purtscher flecken (areas of inner retinal whitening), associated with reduced visual acuity.

When typical retinal findings occur in the complete absence of trauma, the term Purtscher-like retinopathy is used. Purtscher-like retinopathy has been associated with multiple clinical entities, including acute pancreatitis, pancreatic adenocarcinoma, renal failure, preeclampsia and childbirth, connective tissue disorders, crush injury, fat embolism syndrome, long bone fracture, orthopedic surgery, Valsalva maneuver and weight-lifting, lymphoproliferative disorders and bone marrow transplantation, barotrauma, steroid injections in and around the orbit and nasal passages, retrobulbar anesthesia, hemolytic uremic syndrome, cryoglobulinemia, shaken baby syndrome, and DM [9].

DM has also been associated with other retinal pathology, such as central retinal artery occlusion [10], central retinal vein occlusion [11], and optic neuropathy [12]. In one report of 43 patients with polymyositis and DM, only six (14%) had retinopathy [13]. However, when the retina is affected visual prognosis is often poor with an irreversible visual loss [14]. The presence of retinopathy with CWS has also been suggested as a sign of an aggressive subtype of DM [6].

In this case report we describe a case of a 21-year-old woman affected by acute DM associated with retinopathy. A multimodal examination was carried out, which included complete ophthalmoscopic fundus examination, swept source-optical coherence tomography (SS-OCT), fluorescein angiography (FA), and optical coherence tomography angiography (OCTA), allowing us to better understand the etiology and long-term sequelae.

SS-OCT is the latest milestone in retinal and choroidal imaging. With its wavelength of 1050 nm, longer than other spectral domain-optical coherence tomography (SD-OCT 840 nm), it can overcome ocular opacities, such as cataract or vitritis, thus allowing visualization of the retina and choroid in eyes with disabled fundus view [15]. Moreover, SS-OCT offers good visualization of the retina and choroid with a single image acquisition without the use of enhanced depth imaging (EDI) function.

FA gives information about the retinal vasculature, analyzing their permeability to the injected dye.

OCTA is a non-invasive imaging technique that employs motion contrast imaging to high-resolution volumetric blood flow information generating angiographic images. Optical coherence tomography (OCT) angiograms are en face images that can be scrolled outward from the internal limiting membrane to the choroid to visualize the individual vascular plexus and segment the inner retina, outer retina, choriocapillaris, or other area of interest [16].

## Case presentation

A 21-year-old white woman presented to our emergency room with a 1-month history of fatigue, muscle aches, nausea, cutaneous rash, and 5 days of blurred vision in her left eye (OS).

She denied any double vision, pain with eye movements, flashing lights, floaters, or changes in color vision. She had no recent sick contacts or travel. She had no past ocular or medical history. Her family history for ocular or autoimmune disease was unremarkable. She used non-steroidal anti-inflammatory drugs as needed for her recent myalgia.

She presented with a malar rash and some violet raised papules erupting on her elbows and knees that were made worse by exposure to sunlight. Her arterial pressure was 115/70 mmHg. Best corrected visual acuity was 20/20 in her right eye (OD) and 20/100 in her OS.

Intraocular pressure (IOP) was normal in both eyes (OU). Extraocular movements were conjugate and full and she had no subjective red desaturation. Conjunctiva was white without dilated conjunctival vessels, cornea was clear, and anterior chamber showed no evidence of cell or flare, bilaterally.

On dilated examination she had bilateral retinal vascular engorgement, scattered foci of inner retinal whitening consistent with CWS, and some superficial hemorrhages in OU.

**Fig. 1:**
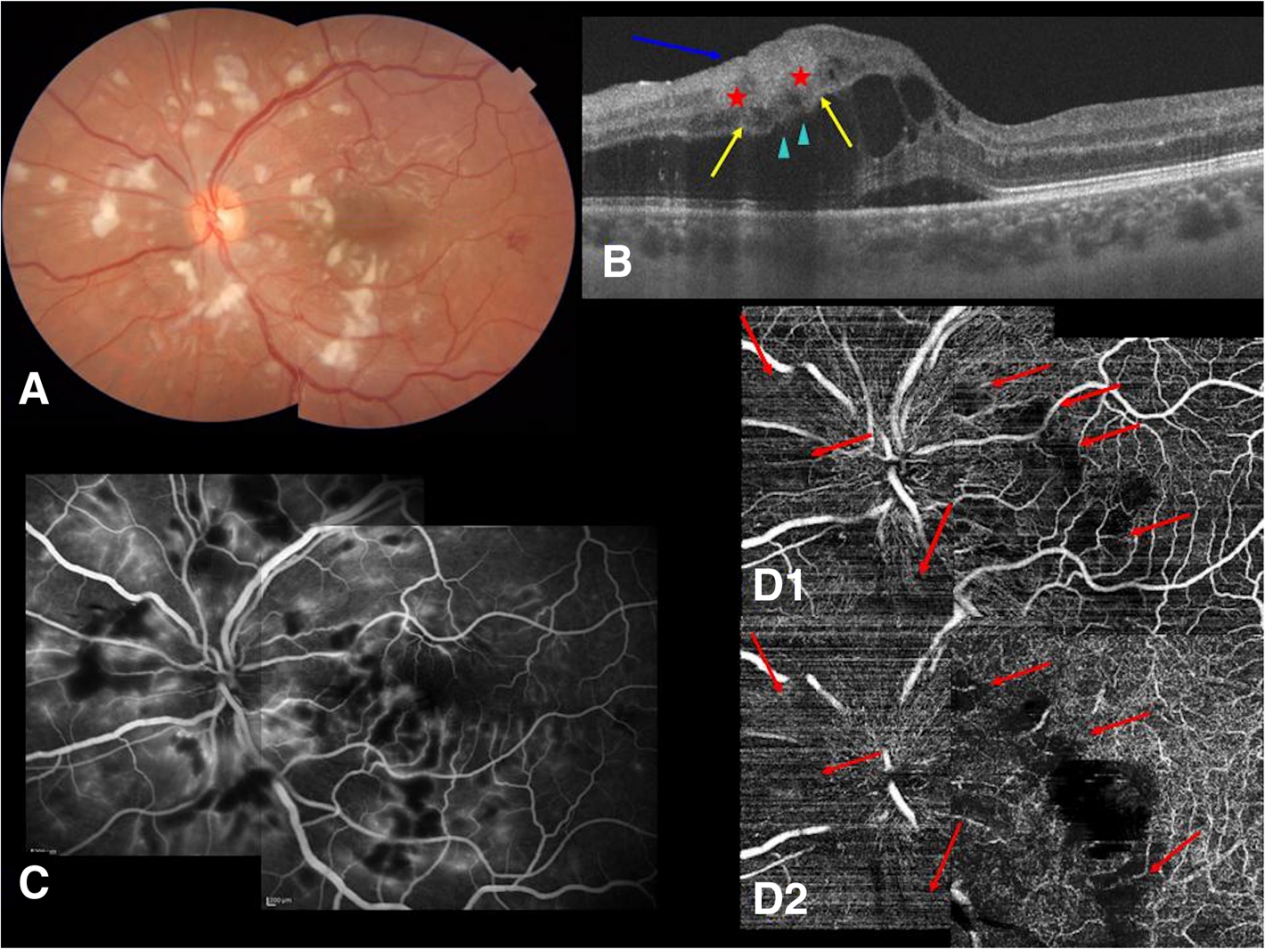
Composite at time of diagnosis in left eye. **a** Fundus color picture: multiple confluent cotton-wool spots around the optic nerve head and the macula; some superficial hemorrhages and moderate diffuse venous dilatations are also visible. **b** Swept source-optical coherence tomography 6 mm scan (*arrowheads for directions*): severe central macular edema with the presence of serous subretinal fluid is shown. Scans passing through cotton-wool spots show homogeneous hyper-reflective fiber layer as result of ischemic edema of nervous layer (*blue arrow*). The edema in a few parts also involves the inner nuclear layer (*yellow arrows*), giving a thickened and hyper-reflective or hypo-reflective appearance. Ganglion cell layer appears hyper-reflective and thickened in a few parts (*stars*). In the retinal areas affected by cotton-wool spots, inner nuclear and outer plexiform layers appeared wavy (*arrowheads*). **c** Fluorescein angiography: vascular walls enhancement, veins dilatation, and capillary occlusion where cotton-wool spots are present. **d** Optical coherence tomography angiography: superficial (*D1*) and deep (*D2*) capillary occlusion in all the areas affected by cotton-wool spots are shown (*red arrows*)

These changes were primarily around the macula and optic nerve and spared the peripheral retina (Fig. 1a).

Optic disks were pink with sharp margins and a cup-to-disc ratio of approximately 0.4.

Triton Plus® SS-OCT (Topcon Corporation, Tokyo, Japan) (Fig. 1b) showed light central macular edema in her OD and severe central macular edema in her OS with the presence of serous subretinal fluid. SS-OCT sections were obtained on the CWS highlighting the presence of hyper-reflective material affecting the nerve fiber layer and reaching even the inner nuclear layer in some sections. This is considered a sign of ischemia-induced edema. Consistent with this edema, inner nuclear and outer plexiform layers appeared wavy and were partially masked by the overlying CWS.

FA (Spectralis HRA+OCT; Heidelberg engineering, Heidelberg, Germany) (Fig. 1c) showed generalized vascular walls enhancement, veins dilatation, and capillary leakage whereas, consistent with CWS, vascular occlusion was observed.

Triton Plus® SS-OCTA (Topcon Corporation, Tokyo, Japan) (Fig. 1d) confirmed superficial and deep capillary occlusion in all areas affected by CWS, but these areas appeared more confluent than in FA. No alterations were visible in outer retina and choriocapillaris OCTA segmentations, but some shadowing effects due to the overlying CWS were present.

At presentation our patient had symptoms that indicated myositis including diffuse muscle pain, weakness, malar rash, and raised papules on elbows and knees. Her muscle enzymes were significantly elevated with a creatine kinase (CK) of 17,030 U/l (normal range 22–198 U/l) and an aldolase (ALDOA) of 106 (normal range 0.5–3 UI/l). Electromyography and nerve conduction study results were suggestive of moderate myositis and a subsequent right thigh muscle (vastus lateralis) biopsy was consistent with an inflammatory myopathy, showing vascular inflammation and a perifascicular atrophy. A myositis antibody panel was positive for an anti-Jo1 antibody. Given the constellation of findings our patient was finally diagnosed as having DM.

Despite the absence of a clear consensus over the most indicated retinal therapy [9], in agreement with a rheumatologist who was managing the systemic disease, treatment was started with 1 mg/kg per day of orally administered prednisone, but she showed no improvement over the course of a week. Therefore, therapy was modified with the administration of a high dose of intravenously administered methylprednisolone (1000 mg daily), showing a slow favorable change after 1 week with a reduction in macular edema and subretinal fluid.

At this point, a decision was taken to start a steroid-sparing immunosuppressive drug, so the corticosteroids were progressively tapered and therapy with cyclophosphamide was started.

The protocol for cyclophosphamide that was used was 500 mg/m^2^ (maximum 500 mg) administered intravenously every 2 weeks for the first three doses and then 750 mg/m^2^ (maximum 1200 mg) every 4 weeks for a total of six doses, with no further cyclophosphamide infusions. Thus, a standardized non-continuous treatment protocol was used, with the cyclophosphamide treatment course completed within 4 months of initiation [17, 18].

After 3 months of therapy, best corrected visual acuity was returned to 20/20 in OU with subjective temporal pericentral scotoma in her OS.

**Fig. 2:**
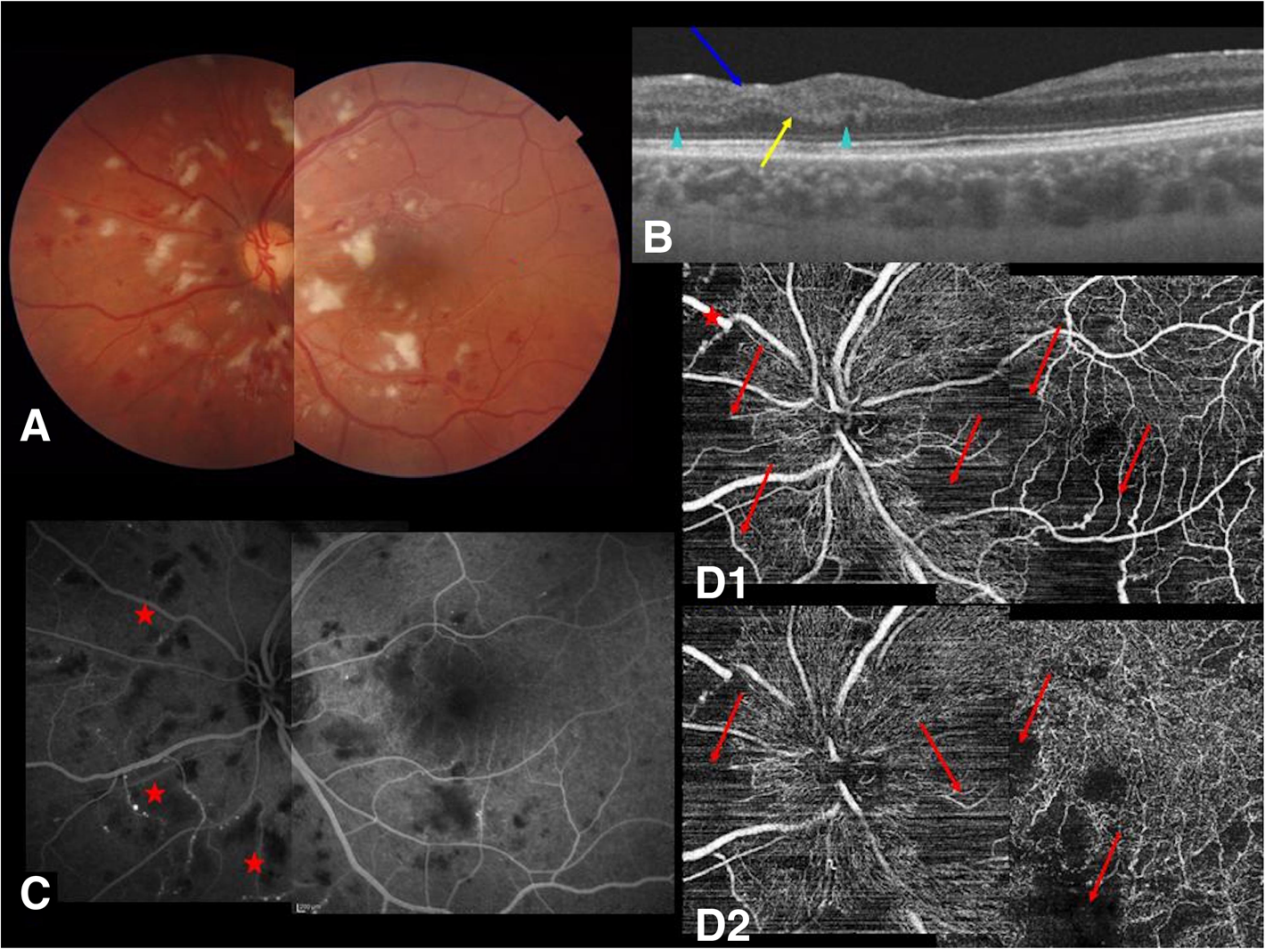
Composite after 3 months of therapy in left eye. **a** Fundus color picture: cotton-wool spots reduced in number and dimensions. New hemorrhages appeared at fundus examination. **b** Swept source-optical coherence tomography 6 mm scan: no edema or subretinal fluid was visible. Ganglion cell layer thinning is shown in all the areas previously affected by cotton-wool spots (*blue arrow*). Inner nuclear and outer plexiform layers conserved their wavy aspect (*arrowheads*). The inner nuclear layer thinned in a few parts (*yellow arrow*). **c** Fluorescein angiography: normal diameters of arteries and veins without wall enhancement are shown. Capillary and venule angioectasias inside ischemic areas previously affected by cotton-wool spots are visible (*red stars*). **d** Optical coherence tomography angiography: superficial and deep capillary occlusions (*red arrows*) and capillary angioectasias (*red stars*) are shown

A funduscopic examination (Fig. 2a) revealed a reduction in number and dimension of the CWS and some new hemorrhages in OU.

SS-OCT (Fig. 2b) showed a complete resolution of the cystoid macula edema and subretinal fluid. The ganglion cell layer and, to a small extent, even the inner nuclear layer were thinned in the areas previously affected by CWS, confirming the ischemic nature of the edema. The wavy appearance of inner nuclear layer and outer plexiform layer was unchanged at this stage.

FA (Fig. 2c) showed normal diameters of arteries and veins without wall enhancement. Consistent with ischemic areas previously affected by CWS, capillary and venule angioectasias were noticeable. However, no retinal neovascularization was visible in OU.

OCTA (Fig. 2d) confirmed the same capillary occlusions visible in FA and it detected venule angioectasias as well.

**Fig. 3:**
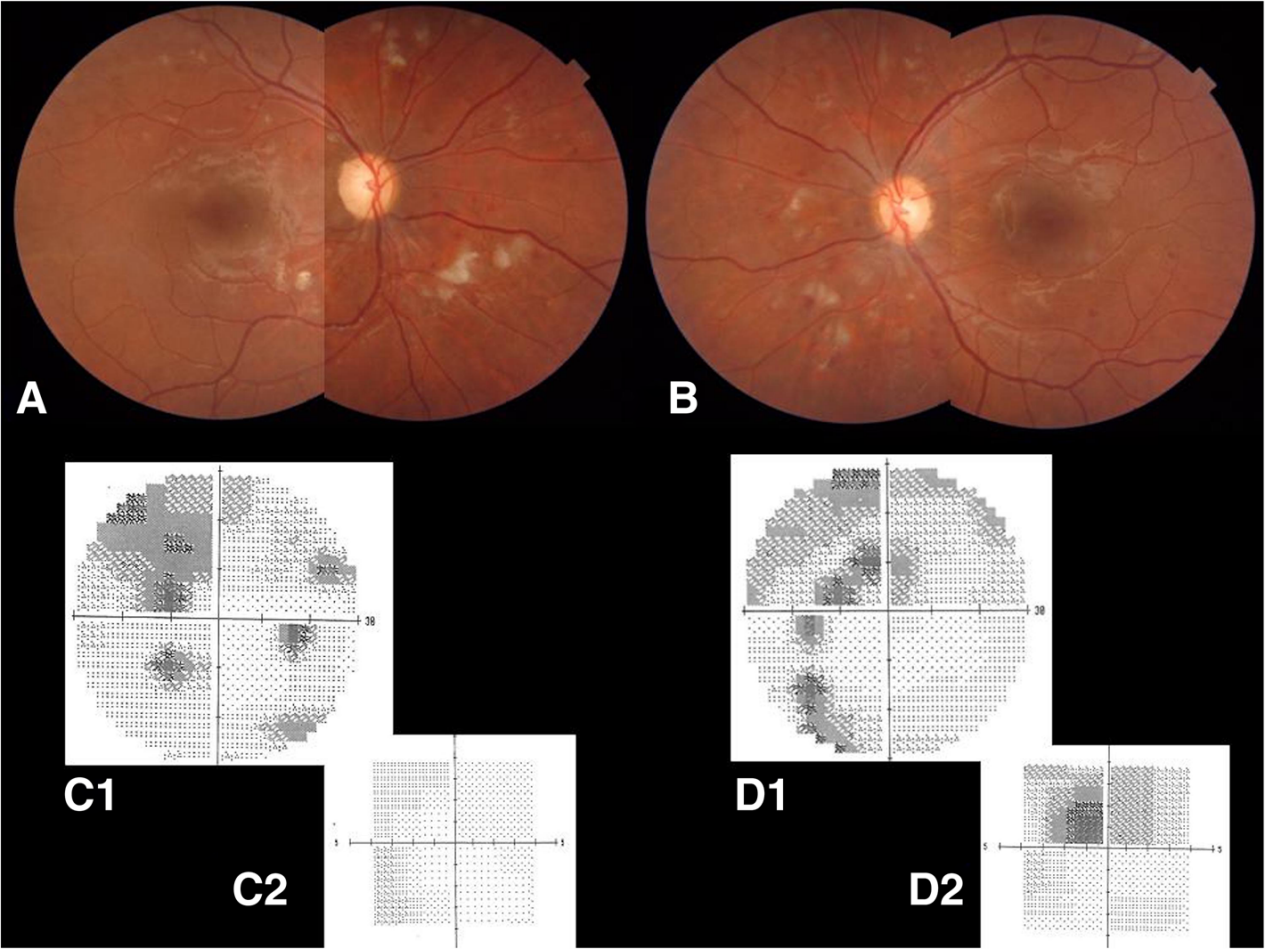
**a**, **b** Color fundus (**a** right eye; **b** left eye) after 6 months of immunosuppressive therapy. Cotton-wool spots and hemorrhages almost resolved. **c**, **d** Visual fields of both eyes (**c** right eye; **d** left eye) assessed at the same time as color fundus. Although cotton-wool spots resolved, visual field defects persist (*C1* and *D1*, 30 degrees; *C2* and *D2*, 5 degrees)

After 6 months of therapy, CWS were almost completely resolved and no new hemorrhages were seen (Fig. 3a,b). Best corrected visual acuity remained stable at 20/20 in OU. However, the visual field examination performed after disease resolution confirmed the presence of multiple and confluent scotomas in OU, with central involvement in OS (Fig. 3c,d).

## Discussion

Multimodal diagnostic evaluation has been used to study Purtscher-like retinopathy in this case of DM. Retinopathy in DM is a rare but a potentially blinding entity. Although macular edema is reversible, ischemic macular involvement can lead to severe irreversible visual impairment.

It is reported that the pathogenesis of Purtscher’s retinopathy and Purtscher-like retinopathy is linked to the formation of several kinds of emboli, such as air, fat, leukocyte aggregates, fibrin, platelets, and complement activation [19].

DM is characterized pathologically by varying degrees of perifascicular atrophy, vasculopathy, and perivascular inflammation [20]. It has been proposed that inflammation and leukocyte aggregates induced by complement activation may occlude the precapillary arterioles in the human retina [19]. This process might also be facilitated by the vasculitis due to the capillary endothelial damage by inflammatory factors [8].

In our patient, SS-OCT, FA, and OCTA scans showed typical vascular inflammatory signs and retinal inflammatory signs at presentation. In particular, OCT scans revealed cystoid macular edema, subretinal fluid, and ischemic edema of the inner retina, FA showed vascular walls enhancement, veins dilatation, and capillary occlusions, whereas OCTA confirmed capillary occlusions and helped define their exact locations.

We are unaware of any report in the literature describing OCTA findings in retinopathy associated with DM. In acute phase, this examination showed both superficial and deep retinal capillary occlusion in the same areas of FA, with ischemic areas more confluent than in FA. It also excluded choriocapillary occlusions.

After the resolution of the inflammatory phase, SS-OCT scans showed inner retina thinning as a result of ischemia, FA showed permanent capillary occlusion, and OCTA confirmed both superficial and deep retinal capillary occlusion. A visual field examination showed permanent scotomas in areas that corresponded to areas that were previously affected by CWS.

These findings help to clarify the potential irreversible visual impairment that can occur even after successful disease resolution, as described in other studies [14]. In fact, if the macula is affected by complete capillary occlusion or by retinal thinning of the inner nuclear layer, visual prognosis remains poor.

Therefore, our suggestion in cases of Purtscher-like retinopathy associated with DM is to come to a correct diagnosis as fast as possible, start a treatment with high doses of intravenously administered glucocorticoids in agreement with a rheumatologist, and consider when to switch to a steroid-sparing regimen with immunosuppressive therapy based on the clinical evolution. The goal is to avoid ischemic damage extension and prevent macular involvement.

## Conclusion

The case presented notes the importance of a complete ophthalmic examination with multimodal imaging in cases of Purtscher-like retinopathy associated with DM. It is essential to come to a diagnosis as fast as possible in order to prevent retinal ischemic extension and macular involvement, which are irreversible complications.

OCTA plays an important role in defining the early ischemic damage and helps to localize the level of the vascular occlusion. This is the first time that OCTA characteristics are described in a case of Purtscher-like retinopathy associated with DM and we believe that OCTA could potentially replace FA in the early assessment and follow up of a Purtscher-like retinopathy.

## Data Availability

All data supporting our findings are provided in the manuscript.

## Abbreviations

CWS: Cotton-wool spots
DM: Dermatomyositis
FA: Fluorescein angiography
OCT: Optical coherence tomography
OCTA: Optical coherence tomography angiography
OD: Right eye
OS: Left eye
OU: Both eyes
SS-OCT: Swept source-optical coherence tomography
